# Charting the onset of Parkinson-like motor and non-motor symptoms in nonhuman primate model of Parkinson’s disease

**DOI:** 10.1371/journal.pone.0202770

**Published:** 2018-08-23

**Authors:** Gourav R. Choudhury, Marcel M. Daadi

**Affiliations:** 1 Southwest National Primate Research Center, Texas Biomedical Research Institute, San Antonio, Texas, United States of America; 2 Research Imaging Institute, Departments of Radiology, Cell Systems & Anatomy, University of Texas Health at San Antonio, Texas, United States of America; Hudson Institute, AUSTRALIA

## Abstract

Parkinson’s disease is a progressive neurodegenerative disease increasingly affecting our aging population. Remarkable advances have been made in developing novel therapies to control symptoms, halt or cure the disease, ranging from physiotherapy and small molecules to cell and gene therapy. This progress was enabled by the existence of reliable animal models. The nonhuman primate model of Parkinson’s disease emulates the cardinal symptoms of the disease, including tremor, rigidity, bradykinesia, postural instability, freezing and cognitive impairment. However, this model is established through the specific loss of midbrain dopaminergic neurons, while our current knowledge reflects the reality of Parkinson’s disease as a multisystem disease. Parkinson’s disease involves both motor and non-motor symptoms, such as sleep disturbance, olfaction, gastrointestinal dysfunctions, depression and cognitive deficits. Some of the non-motor symptoms emerge earlier at the prodromal phase and worsen with disease progression, yet in basic and translational studies, they are rarely considered as endpoints. In this study, we set to characterize an ensemble of less described motor and non-motor dysfunctions in the marmoset MPTP (1-methyl-4-phenyl-1,2,3,6-tetrahydropyridine) model. We provide evidence that this animal model expresses postural head tremor and a progressive worsening of fine motor skills, movement coordination and cognitive abilities over a 6-month period. We report for the first time a non-invasive approach showing detailed analysis of daytime and nighttime sleep and circadian rhythm disturbance remarkably similar to Parkinson’s disease patients. This study describes the incidence of tremors, motor and non-motor dysfunctions in a preclinical model and highlights the need for their consideration in translating effective new therapeutic approaches for Parkinson’s disease.

## Introduction

Parkinson’s disease (PD) is a progressive neurodegenerative disease increasingly affecting our aging population. Risks for developing PD include increased life expectancy, environmental exposure to toxins, genetic susceptibility, life style and brain injuries. Tremendous advances have been made in developing novel therapeutic approaches to halt the disease ranging from small molecules to cell and gene therapy [1]. These advancements and their further development depend on a variety of reliable animal models including small organisms, rodents and nonhuman primates (NHP), either chemically induced or genetically engineered [2]. Multicellular organisms such as C. Elegans [3–5], drosophila [6, 7] and zebrafish [8–10] and rodent genetic models [11–13] have been playing an instrumental role in advancing our understanding of PD and Parkinsonism in general. In translational research, the NHP 1-methyl-4-phenyl-1,2,3,6-tetrahydropyridine hydrochloride (MPTP)-lesioned model is still considered the gold standard for modeling some of the complex features associated with PD, which are impossible to model in other species [2, 14–21]. The model’s authenticity is primarily based on its neuroanatomical, immunological and physiological similarities with humans. The NHP MPTP model amazingly replicates the cardinal symptoms of PD including rigidity, bradykinesia, akinesia, tremor, postural instability, freezing and cognitive impairment. Importantly, MPTP demonstrates common causative mechanisms of PD as seen in humans, including the inhibition of complex I of the electron transport chain in the mitochondria, perturbation of the immune system and α-synuclein expression in the midbrain DA neurons, although without Lewy body formation [22–25].

The NHP MPTP model is well established through the specific loss of midbrain DA neurons; however, our current knowledge of PD and Parkinsonism indicate that PD is a multisystem disease [26–31]. PD involves motor (MS) and non-motor symptoms (NMS), which correlate with the neuropathology and the implication of various neurotransmitter systems [27, 32, 33]. To date most translational studies primarily address the loss of midbrain DA neurons and consider solely hallmark MS as the major outcome for efficacy. Animal models have more to offer. It is more important than ever and essential to take a closer look at disease development and progression, and to establish sensitive and rigorous endpoints with relevancy to our understanding of disease mechanisms and preclinical development. It is rare that postural tremor, cognitive deficits, sleep disturbance or circadian rhythm are considered as outcome measures in animal models, yet these symptoms may appear quite early on and become disabling for PD patients. By considering these symptoms in research and development, the wide spectrum of disabilities that accompany PD will be adequately addressed.

In the present study, we specifically address motor and non-motor deficits, including tremor, cognitive deficits, sleep disturbance and changes in circadian rhythm in the NHP MPTP model. Routine analysis of these parameters might address the unmet needs in translating effective therapies for PD.

## Materials and methods

### Ethics statement

All experiments were performed on marmosets (Callithrix Jacchus) (n = 3) from the Southwest Nonhuman Primate Research Center (SNPRC) colony. All the procedures were performed in strict accordance with the recommendations proposed in the Guide for the Care and Use of Laboratory Animals, National Research Council U. S. A. The protocols were approved by the Institutional Animal Care and Use Committee for Texas Biomedical Research Institute (approval no. 1461CJ, 1469CJ). All nonhuman primates held and used within the SNPRC program of care at the Texas Biomedical Research Institute are maintained under conditions that meet USDA Animal Welfare Regulations, OLAW standards, and National Institute of Health (NIH) guidelines as stated in the *Guide for the Care and Use of Laboratory Animals* (81h Edition, 2010), NAS-ILAR recommendations, and AAALAC accreditation standards for these species. Texas Biomed, including the SNPRC as a component of its overall program, is fully accredited by AAALAC International. The center promotes social housing caging, with structural complexities for environmental enrichment with the detailed observation of ongoing animal activities. The temperature inside animal quarters is maintained at 80°F and humidity (60%) suitable for marmosets. Animals are fed constant nutrition, complete life-cycle commercial monkey chows, supplemented daily with fruits and vegetables, and municipal drinking water is available at all times. All research activity has been conducted in accordance with the IACUC oversight process. The SNPRC employs a large number of full-time professional staff members to provide expertise in program administration, animal husbandry, clinical medicine, psychological well-being, facilities maintenance, animal records, and technical research support. All procedures were performed to minimize discomfort, distress or pain. When necessary sedation and anesthetic agents are used to render the animal unconscious and therefore insensate to handling, discomfort, or pain. Likewise, when necessary analgesics are used to reduce any potential pain. All animals are enrolled in the environmental enrichment program. Enrichment provided to the animals consists of social contact, structural enrichment (e.g., perches, swings), manipulable enrichment (e.g., chew toys, balls), nutritional enrichment (e.g., fruit, grain), sensory enrichment (e.g., television, radio), and occupational enrichment (e.g., food puzzles). All enrichment provided is documented, and any deficiencies are addressed. Although not performed in this study, the veterinarians at the SNPRC perform humane euthanasia of animals and in accordance with the professional principles and practices specified by the *American Veterinary Medical Association Guidelines for the Euthanasia of Animals*: *2013 Edition*. Animals destined for euthanasia are injected intraperitoneally with sodium pentobarbital overdose (100 mg/Kg) followed by transcardiac perfusion with phosphate buffered saline and 4% paraformaldehyde for tissue processing.

### MPTP dosing regimen

MPTP (Sigma Aldrich,) was dissolved in physiological saline (0.9% NaCl) at a concentration of 2mg/ml. The injection of MPTP was conducted in a special biohazard room under negative-pressure and the personnel involved wore personal protection equipment. The MPTP was subcutaneously injected (2mg/Kg B.wt) for 5 consecutive days [34]. After a wash out period of 72 hours from last MPTP injection, the marmosets (n = 3) were returned to their home cages and monitored twice daily for rest of the study period.

### Parkinson’s disease rating scale (PDRS)

The severity of Parkinson-like symptoms in the marmosets was categorized using a validated parkinsonian rating scale for NHP [35]. The PDRS correlates highly with striatal dopamine concentrations detected by postmortem immunohistopathology [36] and it is modeled on the Unified Parkinson’s Disease Rating Scale (UPDRS) [37] and the Hoehn & Yahr scale used clinically to categorize PD patients [38]. The PDRS was performed in daylight by video recording animals for 30 minutes. The evaluation was carried out biweekly before and after MPTP injections. The videos were scored by a blinded operator using PDRS, with a maximal disability score of 57 in the following manner: 0 = normal, 1 = mild, 2 = moderate, 3 = severe; rest tremor, action tremor, tremor of the head, tremor right arm, tremor left arm, freezing, locomotion, fine motor skills right hand, fine motor skills left hand, bradykinesia right arm, bradykinesia left arm, posture, hypokinesia, balance, posture, startle response, gross motor skills right hand and gross motor skills left hand, apathy (defined as a state of indifference), vocalization, drooling or frothing, tongue/face/lips. Independently of the PDRS, rigidity was assessed by evaluating the resistance to passive joint movements and the range of motion during reaching for food. Prior to MPTP lesion, we trained animals using their favorite food (i.e. marshmallows) as reward. The marmosets were trained and conditioned to perform the rewarding visually guided task of reaching and grabbing a marshmallow. The evaluation was performed before and after MPTP.

### Tremor analysis

Video recordings of the animals following the induction of PD were used in the tremor analysis. Postural head tremors were measured by counting the number of bilateral oscillations (per second) of the head from the midline axis (along the center of the head). The action tremors were quantified by recording the number of arm oscillations per second during the performance of the object retrieval task. To verify the accuracy of the measurements, we used a grid on the screen and identified the axis where the oscillation occurs around, similarly to a pendulum, then the video recordings were slowly playbacked (speed reduced by 50%) and analyzed again. Microsoft excel was used to calculate the mean and standard deviation of the quantified data.

### The object retrieval task with barrier detour (ORTBD)

The object retrieval task with a barrier detour is a reward based behavioral testing system that we previously described to evaluate motor and cognitive functions of NHP [39]. Briefly, the task requires the test subject to retrieve a reward (marshmallow) fastened to a tray from the open side (bypassing the barrier) of a transparent box. For the current study, the testing apparatus was modified to fit the marmoset’s home cage and the animals were acclimatized to apparatus prior to testing. Behavioral analysis was done for 3 consecutive days with 20 trials per day before and after administration of MPTP. All parameters measured were previously described in detail [39]. During each trial the orientation of the open side of the box was randomly changed to either left or right of the animal or towards the opening of the cage. The entire process was recorded using a video camera and the recordings were later used for scoring and data analysis. During each trial, the following responses were scored (1) ability of the animal to reach the front, left, or right side of the box, scored under the term “reach act”; (2) hand of choice for the reach, either left or right, scored under the term “hand used”; (3) the outcome of the reach, either success or failure, scored under result section.

Using the above parameters, we were able to analyze additional variables: 1) Reaching disability: Reaching into the open side of the box but without retrieving the reward. 2) Movement initiation time: Latency from the screen being raised to the subject touching the box or reward. 3) Execution: Retrieving the reward from the box on the first reach of the trial (indicates competence on the task). 4) Correct: Eventually retrieving the reward from the box on the trial (>1 reach on the trial to retrieve the reward because unlimited reaches per trial were allowed). 5) Reach number: Number of times the animal made an attempt and touched the box. 6) Hand preference: Hand (left or right) subject used for the first reach of the trial. 7) Hand bias: Total number of left and right hand reaches on each trial. 8) Awkward reach: Reaching with the hand farthest away from the box opening (either the left or right side). 9) Perseverative response: Repeating a reach to the side of the box that was previously open but then closed. 10) Barrier reach: Reaching and touching the closed side of the test box. The results from the data analysis were plotted using Graph pad prism statistical software.

### Activity and sleep analysis

The diurnal behavior of the marmosets was monitored using the actiwatch mini (Cam*n*tech, UK). Actiwatch mini is an accelerometer device that measures the intensity of the test subject’s omnidirectional movements in units or counts, which is directly proportional to the animal’s activity. The device (2 cm in diameter) was placed on a collar around the neck of the marmoset. The animals were acclimatized to the collar in short sessions of 15 min followed by gradual increments of 30 min, 1hr, 3hrs, 6hrs and 12hrs. Once acclimatized, the actiwatch mini was attached to the collar and the activity-rest data was recorded for a period of 24 hours on 3 separate days before and after MPTP (1 and 6 months) administration. The actiwatch was placed on the animal at 8:30 AM and the device was preset to start collecting data at 9:00 AM for the next 24 hrs. The following day the actiwatch was removed after 9:00 AM and the data was transferred to a computer through an actiwatch reader using the actiwatch activity and sleep analysis-7 software (Cam*n*tech, UK). For sleep analysis, the period of sustained quiescence (marmosets sleep cycle) starting at 7:00 PM in the evening to early morning 6:30 AM (approximately 11 ½ hrs.) was analyzed using the sleep analysis-7 software to quantify the sleep quality and wakeful periods. The duration of sleep time was corrected for individual variations in the animal’s behavior to fall asleep at different time of the evening, thereby keeping the period of sleep time analyzed same for all the animals. The analyzed data was then exported to excel and plotted using the Graph pad prism statistical software.

### Nonparametric circadian rhythm analysis (NPCRA)

NPCRA was performed on the Actiwatch data using the sleep analysis-7 software. The following established nonparametric indices of rhythmicity were generated: **IS** (Inter-daily stability) indicates the degree of regularity in the activity-rest pattern of the animal during a 24hr cycle. **IV** (Intra-Daily variability) indicates the degree of fragmentation of activity-rest periods. **L5** (Lowest activity) indicates the average activity level for the least active five hours. **M10** (maximal activity) indicates the average activity level for the most active ten hours. Onset of L5 and M10 indicates the average time of the start of the least active 5-hour period (L5) and the most active 10-hour period (M10) during a circadian cycle and denotes the degree of coordination of individual’s circadian cycle with a normal 24-h cycle. Relative amplitude is estimated by dividing the difference between M10 and L5 period with the sum of M10 and L5. Relative amplitude has a range between 0–1 and higher values indicate a rhythm with higher amplitude. The data was then exported to excel and plotted using the Graph pad prism statistical software.

### Drug treatment

Levodopa (sigma) and carbidopa (sigma) at 1:1 were administered orally once daily mixed in either ensure pudding or cottage cheese or marshmallows at 3–12 mg/Kg B.wt. The drug and vehicle mix was prepared fresh every day and was administered 5 days a week for two consecutive weeks. During the first week of L-Dopa therapy, PDRS and ORTBD were performed on the parkinsonian marmosets (n = 3) 1hr after the administration of vehicle or L-DOPA. Actiwatch analysis was performed during the second week of L-Dopa therapy. For the activity analysis, the actiwatch was setup to start recording at 9:00 AM and the vehicle or drugs was administered at 11:30 AM (after 2.5 hrs of baseline recording). The actiwatches were collected after 24hrs of acquisition, the data was downloaded and analyzed using the sleep analysis-7 software. The activity analysis was performed three times in a week on the three parkinsonian marmosets. The analyzed data was then exported to excel and plotted using the Graph pad prism statistical software.

### Statistical analysis

Statistical analysis was done with Graph Pad Prism statistical software. Significance in differences between 2 groups was performed by applying Student’s t-test where appropriate. For comparison of multiple groups One-Way with Newman-Keuls multiple comparison test or Two-Way ANOVA with Bonferroni post-hoc analysis was performed to identify the significant differences. A P-value of less than 0.05 was considered to be statistically significant.

## Results

### Baseline behavior and activity patterns of the marmosets

Prior to injection of MPTP the marmosets were regularly observed and evaluated to establish a baseline for their behavior. All animals in the group showed constant interactions with each other through a variety of vocalizations.

**Fig 1:**
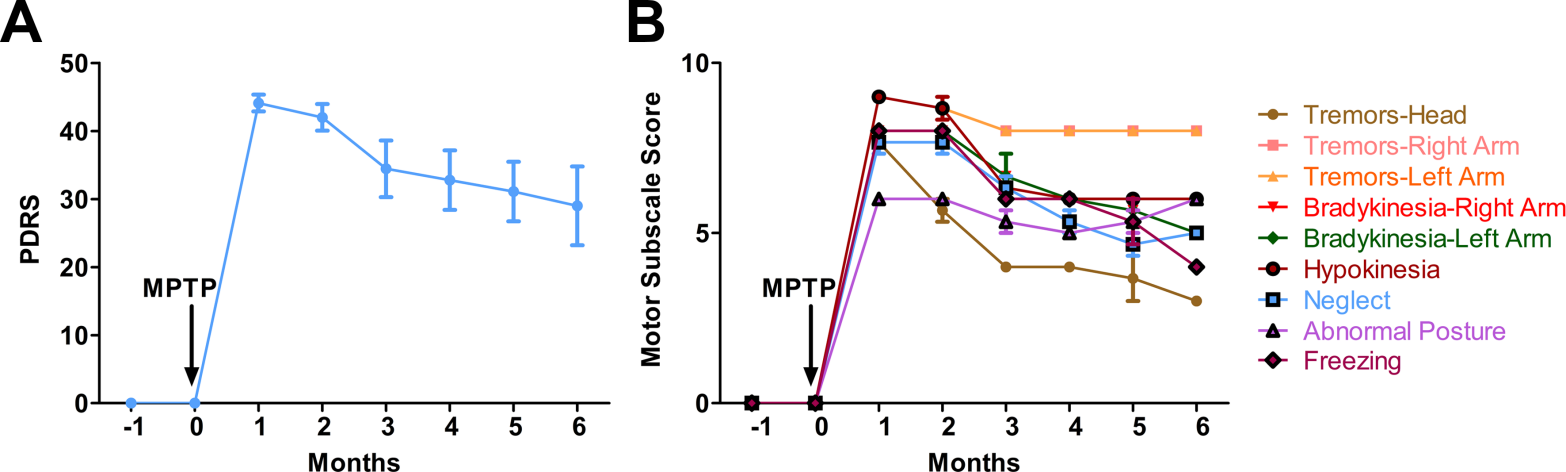
Parkinson’s disease rating scale (PDRS) score of the MPTP-treated marmosets. (A) Monthly cumulative PDRS scores of the parkinsonian marmosets (n = 3) following MPTP injection. The scoring scale is described in detail in the method section. Prior to the injection of MPTP the marmosets showed no evidence of PD disabilities. One month after the injection the marmosets showed severe PD disabilities that gradually stabilized over time. **(B)** Motor subscale scores showing progression of individual parkinsonian disabilities in the MPTP-treated marmosets. Error bars represent standard error.

**Fig 2:**
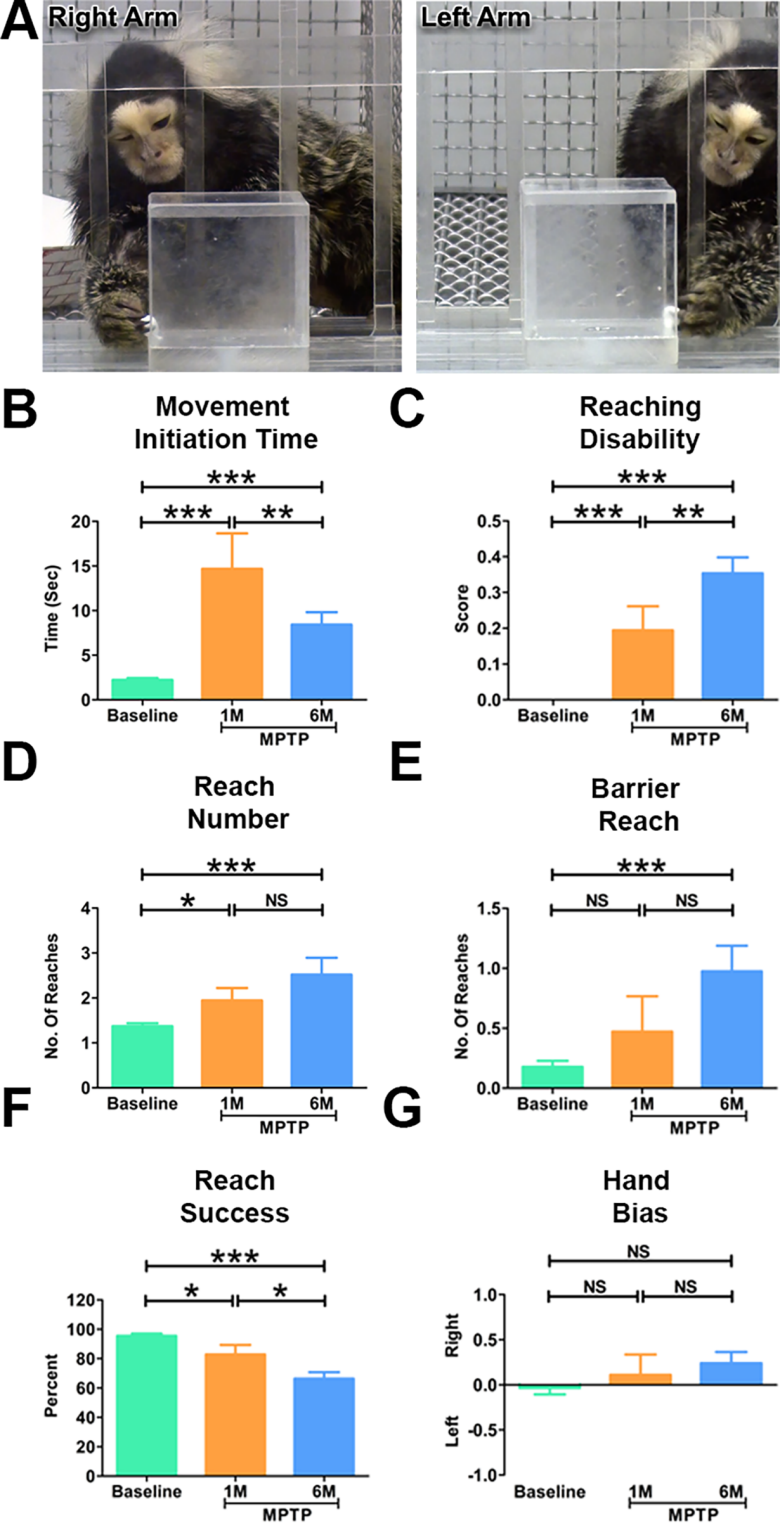
Parkinsonian marmosets demonstrated significant deficits in motor and cognitive functions. (A) Representative images of the marmoset performing the object retrieval task. The marmosets (n = 3) had to learn to by-pass the transparent barrier and reach for the reward through the open side of the box. Quantitative analysis of the object retrieval task before and after (1 month and 6 months) the MPTP administration. (B) Following the induction of PD, the marmosets demonstrated a significantly longer delay in initiating a response (Movement initiation time) during the task. (C) The marmosets demonstrated significantly high reaching disabilities following the induction of PD. (D) The parkinsonian marmosets made significantly more number of attempts to reach the reward during the task. (E) The cognitive task showing that the marmosets made significantly more number of barrier reaches at 6 months. (F) The parkinsonian marmosets were significantly less successful in retrieving the reward during the task. (G) No significant differences were seen in hand bias before and after the induction of PD in the marmosets. Statistical analysis was performed using One-way ANOVA followed by Newman-Keuls Multiple Comparison Test for post-hoc analysis of groups. *P < 0.05, **P < 0.01, ***P < 0.001, NS: Not Significant. Error bars represent standard error.

**Fig 3:**
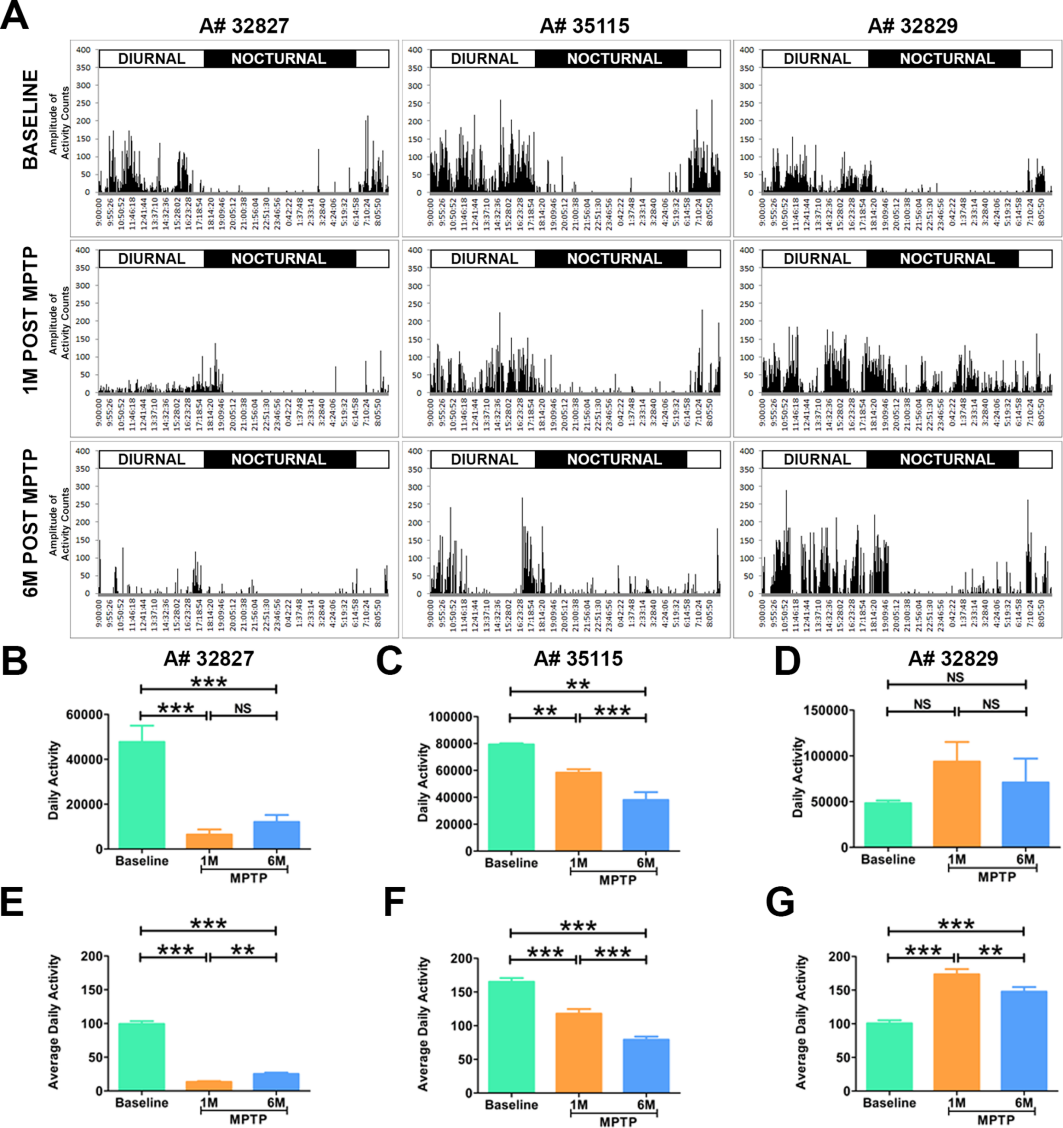
Abnormal diurnal activity in parkinsonian marmosets. (A) Representative actograms of the marmosets before and after the induction of PD. Following MPTP administration the marmosets (n = 3) showed a dramatic change in the pattern of daytime activity. Animals A#32827 and A#35115 showed a decrease in daytime activity, where as monkey A#32829 demonstrated an abnormal increase in activity. Quantitative analysis showed a significant decrease in daytime activity in monkeys (B) A#32827; (C) A#35115. (D) A#32829 showed an abnormal increase in daytime activity at 1 and 6 months after MPTP injection but the difference was not significant. Quantitative analysis of average daily activity showed a significant decrease in (E) A#32827, (F) A#35115, and significant increase in (G). Statistical analysis was performed using One-way ANOVA followed by Newman-Keuls Multiple Comparison Test for post-hoc analysis of groups. **P < 0.01, ***P < 0.001, NS: Not Significant. Error bars represent standard error.

The baseline activity one month prior to MPTP lesion showed no parkinsonian symptoms expressed by the marmosets with the lowest PDRS score (Fig 1). The animals were then evaluated on the object retrieval task with barrier detour to establish a base line for motor and cognitive functions. The marmosets showed no observable deficits in the task and all animals performed to the same degree of competence (Fig 2). We then used the actiwatch device to collect general activity data over 24 hours on 3 separate occasions. The actograms demonstrated a typical diurnal behavior of the marmosets that was consistent among all the animals used in the study (Fig 3). Daytime activity started between 6:30AM & 7:00AM after a prolonged quiescence period (Fig 3A) indicating the end of sleep. Throughout the day, the marmosets generally displayed a biphasic activity with a first peak around 10:00 AM presumably coinciding with feeding that slowly tapered down by noon (Fig 3A). The second peak was seen around 4:00 PM that gradually diminished by 6:00 PM before the animals went into a long period of sustained quiescence (Fig 3A). At night, the actograms showed very little or no activity during sleep.

### The marmosets develop stable parkinsonian syndrome

One month following MPTP injections all the marmosets demonstrated, as expected, severe PD-like symptoms with an average PDRS of 43 (Fig 1). The symptoms included tremors, bradykinesia, and abnormal posture and decreased activity. Tremor is defined based on the clinical classification as previously described in the “Consensus Statement of the Movement Disorder Society on Tremor” [40].

Interestingly, two out of three marmosets displayed characteristic low frequency (4.3 +/- 1.7 Hz) tremor of the head at rest, very similar to symptoms seen in human PD patients (S1 Video). The animal (A# 35115) that did not develop the tremor received a total MPTP dose of 2.88 mg (4 injections 2mg/kg) while the two others (A# 32827, A# 32829) that developed postural tremor receive each a total 4.62 mg and 4.8 mg respectively (5 injections 2mg/kg, SC) [34]. When enticed to initiate a movement by presenting a treat, the marmosets displayed a delay during the initiation of the reaching movement with the manifestation of action tremors during reaching and grasping the treat.

The PD marmosets showed lack of vocalization, they remained silent while the control non-lesioned group vocalizes routinely as usual. This behavior was noticeable during the first 3 months after the MPTP lesion. At 6 months, while one of the marmosets (A# 35115) reinitiated the vocalization, the other two continued to remain silent or vocalized very little compared to healthy animals in the room. The marmosets continued to display resting tremors, bradykinesia, hypokinesia and apathy (defined as a state of indifference) at 6 months (Fig 1) (average PDRS 29) although the severity of motor symptoms was reduced after 6 months, the marmosets did not regain their normal healthy behavior (Fig 1).

### Parkinsonian marmosets demonstrate significant motor and cognitive impairments

Using the object retrieval task, we investigated the motor and cognitive functions before and after the induction of PD in the marmosets. Prior to MPTP injection, the marmosets demonstrated no difficulty in retrieving the reward from the box. As expected, following MPTP injection all animals displayed altered range of motion and bradykinesia with significant increase (p<0.001) in movement initiation time during reaching (Fig 2C). The animals also demonstrated severe action tremor during the intention phase of picking the reward before the movement execution. This severe action tremor was debilitating and led to freezing, significant increase in the number of reaches and reaching disability (as defined in Method section) (Fig 2C anhd 2D). Interestingly, the object retrieval task revealed that the marmosets performed worse at 6 months compared to 1 month indicating the progressive worsening of the fine motor skills and coordination (Fig 2C and 2D).

The detour component in the object retrieval test is a key element of the cognitive skills and problem solving during the skilled action to retrieve the reward [19, 39]. This task involves altering the spatial projection of movement and planning the trajectory of execution to reach around transparent barrier and retrieve the reward. This cognitive task is measured by the barrier reach variable. In an error the animal is unable to perform a detour to retrieve the reward and instead keeps touching the transparent closed side of the box. At one month the MPTP-lesioned animals did not show cognitive deficits measured in barrier reach test. However, six months post-MPTP lesion the marmosets showed significant cognitive impairments (Fig 2E).

### Parkinsonian marmosets displayed abnormal diurnal activity

One month after the MPTP injections, we repeated the actiwatch analysis on the marmosets (n = 3) to investigate the effect of parkinsonian symptoms on marmosets’ activity. Actograms revealed noticeable reduction in the diurnal activity of the marmosets that was due to the hypokinesia. Two marmosets (A#32827, A#35115) demonstrated a significantly (p<0.05) reduced activity during the daytime as depicted by decrease in the number and amplitude of peaks in the actogram (Fig 2B–2G). Interestingly, the PD marmosets were falling asleep during day light while in action, hanging on to the cage (S2 Video) suggesting that daytime sleep disturbance may be a reliable outcome measure. In addition to hypokinesia, one marmoset (A#32829) demonstrated intermittent bouts of high intensity postural tremor that manifested by bursts of increased peaks in the actogram. Quantitative analysis of this activity showed significant difference (p<0.05) from the baseline (Fig 3G).

### Sleep disturbance in the parkinsonian marmosets

**Fig 4:**
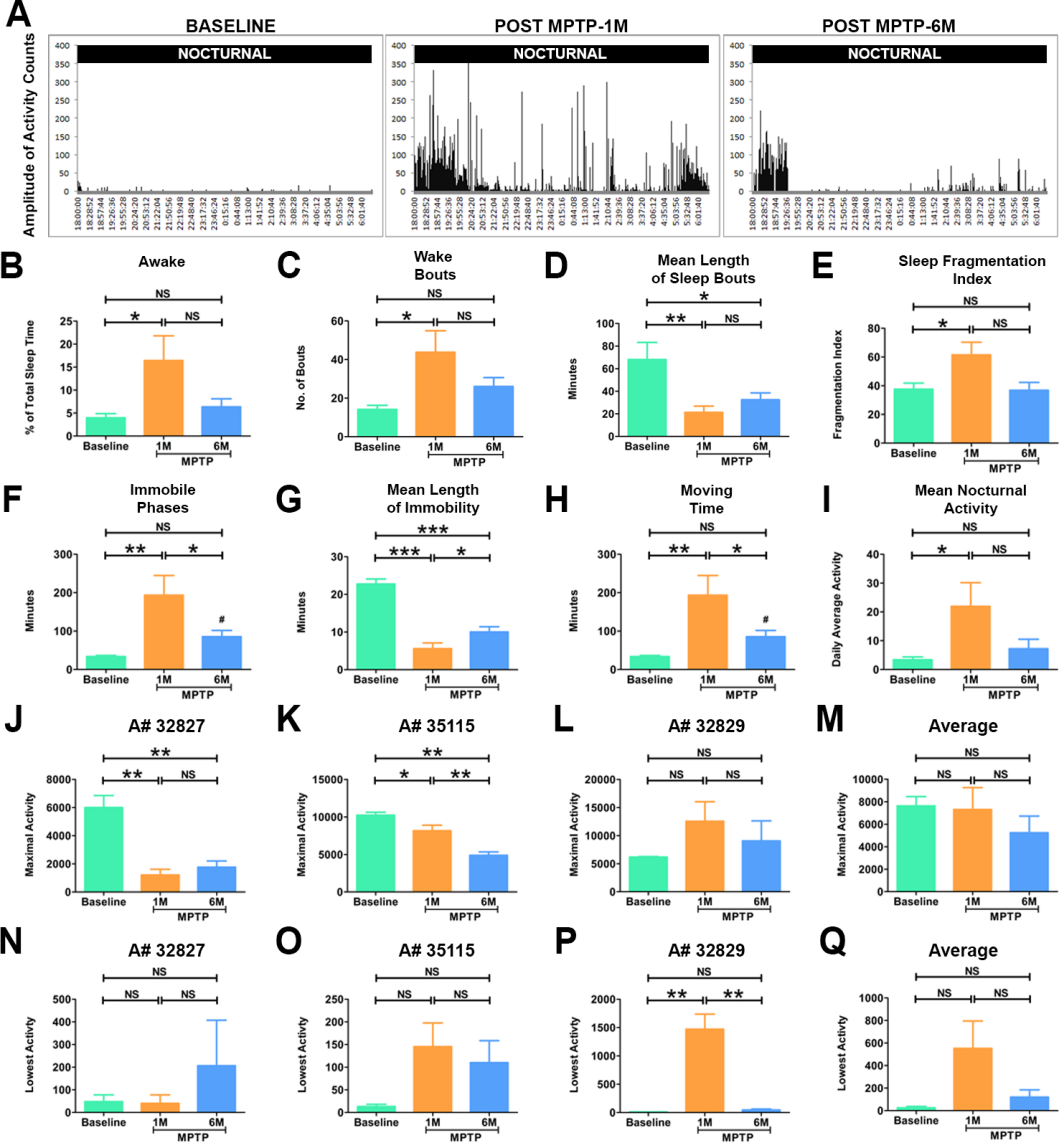
Disturbances in sleep quality in parkinsonian marmosets. (A) Representative actograms of the nocturnal activity of the marmosets before and after the induction of PD. Prior to injection of MPTP the marmosets (n = 3) demonstrated very little activity during night. One month after MPTP injection the marmosets displayed increase in nocturnal activity as depicted in the actogram. The abnormal night activity continued up to 6 months post MPTP administration. (B) Quantitative analysis of sleep quality parameters showed that marmosets demonstrated a significant increase in percent awake time and in (C) the number of wake bouts at 1 month. (D) The mean length of sleep bouts was significantly shorter at 1 month and 6 months after MPTP administration compared to the baseline. (E) The fragmentation index, which indicates the quality of sleep, was also significantly increased at 1 month after MPTP administration. (F) The animals also demonstrated significantly higher number of immobile phases at 1 month. (G) The mean length of immobility that is the time marmosets spent immobile during sleep was significantly shorter at 1 month and 6 months of MPTP administration. (H) The total amount of time animal spent moving at night was significantly increased at 1 month. (I) The mean nocturnal activity was significantly higher at 1 month in the marmosets. (J-Q) Show altered circadian rhythm parameters in the parkinsonian marmosets. Quantitative analysis of the average activity level for the most active ten hours (MA) demonstrated a significant decrease at 1 month and 6 months in monkeys (J) A#32827, (K) A#35115. No significant differences were seen in (L) A#32829. (M) Combined average activity for the MA ten hours (maximal activity) of the marmosets (n = 3) before and after the administration of MPTP showed no statistically significant differences. Quantitative analysis of the average activity level for the least active five hours (LA) showed no significant differences in (N) A#32827, (O) A#35115. However, (P) A#32829 showed a significant increase in activity at 1 month after MPTP injection. (Q) Combined average activity for the LA ten hours (lowest activity) of the marmosets (n = 3) before and after the administration of MPTP showed no statistically significant differences. Statistical analysis was performed using One-way ANOVA followed by Newman-Keuls Multiple Comparison Test for post-hoc analysis of groups. *P < 0.05, **P < 0.01, ***P < 0.001, NS: Not Significant. Error bars represent standard error.

The actiwatch enabled us to non-invasively and quantitatively analyze the activity at night and the soundness of sleep in the parkinsonian marmosets (n = 3). The representative actogram in Fig 4A shows a clear increase in the amplitude of the activity peaks seen at nighttime compared to before the MPTP lesion. Quantitative analysis demonstrated significant increase (p<0.01) in wakefulness and activity at night and in time spent moving (Fig 4A) which remained stable at 6 months. Interestingly, the actogram shows high activity in comparison to the baseline during the time period when animals are supposed to be falling asleep (Fig 4A, 6PM to 8PM) at 1 month and 6 months post-lesion. To investigate the soundness of sleep in the parkinsonian marmosets, we analyzed the mean length of sleep bouts. The results showed that length was significantly shorter (p<0.05) in parkinsonian marmosets both at 1 and 6 months (Fig 4D) suggesting the marmosets woke up more often at night. Moreover, the number of immobile phases at night increased while the length of immobility during sleep significantly decreased (p<0.01) (Fig 4F and 4G) suggesting that PD marmosets were moving during sleep. Together these results suggested, as in PD patients, the parkinsonian marmosets experience abnormal irregular sleep.

### Altered circadian rhythm parameters in parkinsonian marmosets

Given the significant disturbance in sleep, we next investigated whether there is a change in circadian rhythm in the parkinsonian marmosets (n = 3). Non-parametric circadian rhythm analysis demonstrated no significant differences in the degree of regularity in the activity-rest patterns (inter-daily stability) and fragmentation of activity-rest periods (inter-daily variability). However, the activity level for the most active ten hours (MA) was significantly reduced and its onset during the day was delayed at 1 and 6 months (Fig 4J and 4K) (Table 1). We then looked at the average activity level for the least active five hours (LA). One marmoset (A#32829), with severe parkinsonism showed a significant increase in LA at 1-month post MPTP while the other 2 animals showed a trend towards increase in LA. Similar to MA, the onset of LA showed a shift in the parkinsonian marmosets from ~9:00 PM to ~8:00 PM while the amplitude did not significantly change (Table 1). This finding is consistent with the sleep analysis demonstrating delay in sleep-onset insomnia and reveal disturbances in the circadian rhythm.

**Table 1:** Circadian rhythm parameters of the parkinsonian marmosets.

** **	**Baseline**			**1M Post MPTP**			**6M Post MPTP**		
**Animal #**	32827	32829	35115	32827	32829	35115	32827	32829	35115
**IS**	1	0.97 ± 0.03	1 ± 0.004	0.48 ± 0.19	0.94 ± 0.15	0.96 ± 0.09	0.8 ± 0.19	1	1
**IV**	0.881 ± 0.25	1.01 ± 0.29	0.87 ± 0.30	1.65 ± 1.00	1.24 ± 0.47	0.71 ± 0.15	1.6 ± 0.4	1.38 ± 0.87	0.89 ± 0.08
**LA**	47 ± 51	10 ± 1.4	13 ± 8.7	40 ± 64.96	1470 ± 464.2	145 ± 90.4	206 ± 347	45.3 ± 30.89	110 ± 83.8
**LA onset**	21:40 ± 1:00	21:00	22:00 ± 1	20:20 ± 1:31	20:40 ± 0:34	20:00 ± 12	17:00 ± 7:56	21:00 ± 1:43	20:40 ± 2
**MA**	60002 ± 1474.4	6169 ± 157.7	10231.67 ± 661.9	1208 ± 713.5	12553 ± 60.25	8157.6 ± 1277	1768 ± 754	9063 ± 6165	4880 ± 809
**MA onset**	7:00	8:00	7:40 ± 0:34	9:40 ± 0:34	10:00	8:40 ± 0:34	9:40 ± 2:04	8:20 ± 1:31	7:20 ± 1:31
**Amplitude**	5955 ± 1441.6	6159 ± 159.1	10218.67 ±6 54.7	1168 ± 649.08	11083 ± 5653.6	8012 ± 1215	1562 ± 580	9018.3 ± 6192	4770 ± 837
**Rel Amp**	0.985 ± 0.01	0.99 ± 0.007	0.99 ± 0.001	0.96 ± 0.06	0.78 ± 0.56	0.96 ± 0.02	0.857 ± 0.2	0.97 ± 0.03	0.95 ± 0.03

The nonparametric indices of rhythmicity used in the circadian rhythm analysis are described in detail in the Method section. **IS** = Inter-daily Stability; **IV** = Inter-daily variability; **LA** = Lowest activity; **MA** = Maximal Activity; **Rel AMP** = Relative amplitude.

### Effects of L-DOPA therapy

**Fig 5:**
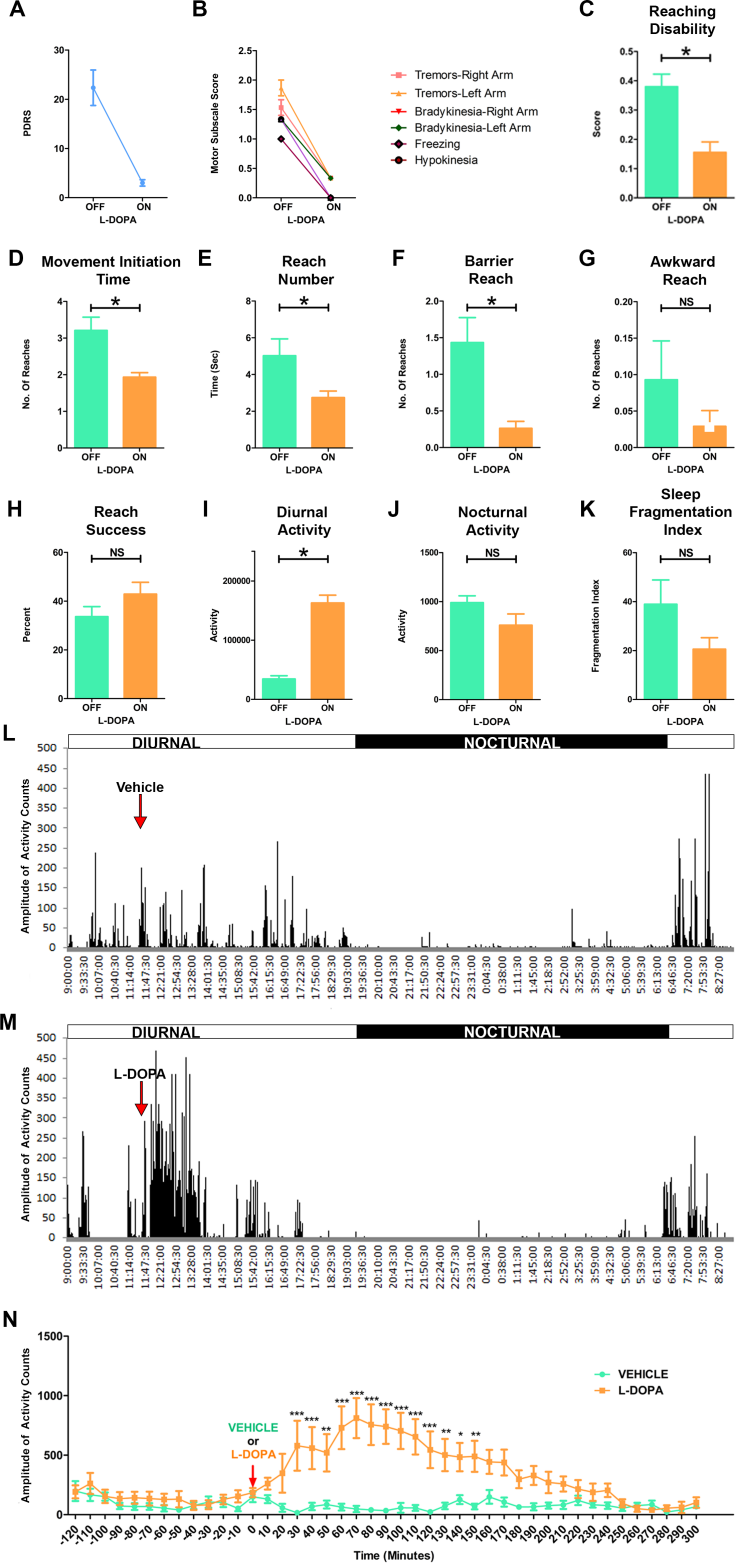
L-DOPA administration improved symptoms and motor deficits of PD marmosets. (A) Cumulative PDRS scores of the marmosets during the on and off L-DOPA treatments. The parkinsonian marmosets (n = 3) showed improvement in PD disabilities following L-DOPA treatment (B) Motor subscale scores showing progression of individual parkinsonian disabilities during on and off L-DOPA treatments. (C-H) Quantitative analysis of the object retrieval task with barrier detour (ORTBD) in the parkinsonian marmosets during on and off L-DOPA treatments. The parkinsonian marmosets showed a significant reduction in (C) Reaching disability, (D) Movement initiation time, (E) Reach number, and (F) Barrier reach. (G) Awkward reach. (H) The PD marmosets showed more reach success on the ORTBD with L-DOPA treatment. (I) Quantitative analysis of total diurnal activity demonstrated a significant increase activity with L-DOPA compared to vehicle. Analysis of sleep demonstrated no significant decrease in (J) Nocturnal activity nor (K) fragmentation index. Representative actograms of the PD marmosets with (L) vehicle and (M) L-DOPA. Arrows indicate the time of administration of vehicle or L-DOPA to the animals. L-DOPA treatment resulted in a marked increase in motor activity of the parkinsonian marmosets. (N) Quantitative analysis of the motor activity of the parkinsonian marmosets with vehicle and L-DOPA. Approximately 30 minutes after the administration of L-DOPA the parkinsonian marmosets begin to demonstrate significant increase in activity, which peaked at 60 minutes. For comparison between two groups *student t-test* was used. Statistical analysis for locomotor activity was performed using Two-way ANOVA followed by Bonferroni posthoc analysis. ***P < 0.001, **P < 0.01, *P < 0.05. Error bars represent SEM standard error of the mean.

We next investigated the effect of L-DOPA treatment on PD-like symptoms, ORTBD and activity of the MPTP lesioned marmosets. One hour after the administration of L-DOPA/Carbidopa or vehicle the PD marmosets (n = 3) were evaluated using the PDRS and ORTBD test. As expected, the PD marmosets exhibited a striking improvement in the general parkinsonian symptoms 1hr after oral administration of L-DOPA (Fig 5A & 5B). The NHP on L-DOPA demonstrated an increase in locomotor activity and improvements in both posture and balance. A reduction in rigidity and bradykinesia was noted as the animals reached and grabbed the treats with ease (S3 Video). Interestingly, animals that stopped vocalizing following MPTP injections returned to vocalizing under L-DOPA treatment. In the ORTBD, the improvements in range of motion and bradykinesia following L-DOPA treatment caused a significant reduction (p<0.05) in reaching disability and movement initiation time compared to vehicle treatment (Fig 5C and 5D). The PD marmosets on L-DOPA made significantly less (p<0.05) number of total and barrier reaches (Fig 5E & 5F). The L-DOPA treatment showed no improvement in the successful attempts to retrieve the reward (Fig 5G & 5H).

Next we investigated the effect of L-DOPA treatment on daily activity of the animals. Shortly after the administration of the L-DOPA, the actograms demonstrated a marked increase in the amplitude and frequency of the movements (Fig 5M). Analysis showed that the activity significantly increases 30 min after the administration of L-DOPA, peaks at 70 minutes and lasts up to 4 hrs (Fig 5M and 5N). In contrast vehicle administration did not show any increase in activity of the PD marmosets (Fig 5L and 5N). Further the total diurnal activity in the PD marmosets was significantly high (p<0.05) with L-DOPA treatment (Fig 5I) compared to vehicle. Analysis of the nocturnal phase showed no effects of L-DOPA compared to vehicle (Fig 5J). Although there was a trend in decrease in sleep fragmentation, L-DOPA treatment did not have significant effect on the sleep quality parameters (Fig 5K).

## Discussion

The present study demonstrates that the NHP MPTP model of PD exhibits significant non-motor Parkinson-like symptoms in long-term follow up. Although there was an improvement in the PDRS, the NHP continued to display motor deficits including postural and action tremors, altered range of motion during reaching and bradykinesia. Importantly, we observed a progressive worsening of non-motor dysfunctions, including cognitive deficits, sleep and circadian rhythm disturbances. L-DOPA treatment improved motor and some of the non-motor dysfunctions. The evidence suggests worsening of fine motor skills, movement coordination and cognitive abilities from 1 to 6 months.

PD patients experience a range of MS and NMS that are currently not fully understood nor rigorously addressed or phenocopied in animal models. While MS manifest later in the disease, when dopamine loss in the striatum exceed the 60% to 80% clinical threshold [41], certain NMS such as sleep disturbance, olfaction and gastrointestinal dysfunctions emerge earlier at the prodromal phase of PD [31, 42, 43] and may serve as early biomarkers. Nevertheless, NMS are sustained and worsen with the disease progression [44–46]. Cognitive and sleep disturbance clearly impact the quality of life of PD patients; however, they are rarely considered as endpoints in pivotal translational studies [43, 47]. Thus, charting the onset and evolution of these motor and non-motor dysfunctions in NHP and other animal models is much needed to better understand the common pathophysiology and symptomatology between human and NHP model of PD and improve translational research.

Compelling evidence suggest that extra-striatal dopaminergic cell body loss and denervation in various structures, including prefrontal cortex, thalamus, globus pallidus, hypothalamus, locus coeruleus, subthalamic nucleus, ventral tegmental area, periaqueductal gray and retrorubral nucleus are affected in PD patients and NHP MPTP models suggesting dopaminergic and non-dopaminergic contribution to non-motor dysfunctions [18, 30, 31, 48–52]. There is also strong evidence of other non-dopaminergic systems involved in the pathophysiology and onset of NMS in patients and animal models [42, 50, 53–55]. These systems include structures like the locus coeruleus, raphe nucleus, nucleus basalis of Meynert, pedunculopontine nucleus, which together involve the norepinephrinergic, serotoninergic and cholinergic systems [27, 28, 31, 50, 53–60]. We have observed that the cognitive deficits measured using the object retrieval with barrier detour appear gradually and reached significance at 6 months post MPTP lesion. These findings are consistent with early studies in NHP models [17, 19, 61] and demonstrate that cognition is a debilitating NMS that develops progressively and a key endpoint to consider monitoring during treatments.

In this study, two out of three PD marmosets developed postural tremor of the head as well as action tremors. The frequency (less than 5Hz) postural head tremor at rest observed is one of the cardinal symptoms that differentiate PD from other forms of parkinsonism [62]. It is infrequent and absent in 10% to 30% of idiopathic PD [62, 63]. Similarly to humans, not all MPTP lesioned NHP develop postural head tremor at rest [64] and given its uncommonness in NHP, we have provided a video (S1 Video) showing an example of this tremor in the marmoset model, which disappeared during movement. Modeling this tremor has been reported in certain species, such as the African Green monkey [65] but not in rhesus macaques [66], squirrel monkeys [67] or marmosets [68]. The lack of systematic occurrence of this type of tremor in the MPTP NHP model was attributed to species differences, route and schedule of MPTP lesion and neural circuits affected by the lesion [16, 69–71]; however, the mechanism behind the genesis and intermittence of this tremor, whether resting or postural remains to be elucidated. In our study, the animal that did not develop the postural head tremor at rest received less MPTP (cumulative dose of 8 mg/kg) than the other two that developed it (cumulative dose 10 mg/kg). These data suggest that the dose may be a contributing factor in the development of this type of tremor and the low dose animal may develop it with additional MPTP dosing, although further studies are needed to specifically address this question. In support of this possibility, patients that took synthetic heroin contaminated with MPTP had a similar effect and showed an evolution with the disease progression of tremor at rest spreading from arm to ipsilateral leg then to all limbs [70].

The parkinsonian marmosets described in this study showed a significant sleep disturbance remarkably similar to PD patients. There was significant increase in wake bouts, moving time, decrease in immobility phase at nighttime and delay in sleep-onset similar to the insomnia and sleep disturbance experienced with PD patients [27, 72]. In daytime, animals frequently napped and recorded falling asleep while engaged with the experimenter, which suggests the occurrence of the sudden-onset sleep observed in PD patients [73, 74]. We report a non-invasive approach revealing a detailed analysis of sleep disturbance. Our findings are consistent with previous studies using electroencephalographic recording in the NHP MPTP model [75–79] showing frequent awake bouts, deregulation of sleep-awake pattern and increased daytime sleepiness. Related to sleep is the circadian rhythm, which we reported as affected in the NHP MPTP model. We noted an inter-individual variability in the maximal and lowest activities in circadian rhythm analysis. This caused the non-significance observed on the effects of MPTP when data were averaged over the 3 animals. This data suggest that similarly to humans, there is inter-individual NHP variability in responses to treatment and that our n = 3 is low to detect these changes in a group. Our analysis of individual changes before and after MPTP led to pertinent information about the model; however, in translational studies aimed at testing efficacy, a larger number of animals may be considered. We observed a significant delay in the onset of the animal’s most active period during the day, and of the most inactive period at night. This is similar to the debilitating rigidity or hypokinesia experienced by PD patients in the mornings [72]. The sleep behavioral disorder is a major NMS in PD patients that affects quality of life [43, 72, 80, 81] and requires consideration among the multiple systems affected in NHP PD research.

In conclusion, the MPTP marmoset model of PD emulates various types of Parkinson-like motor and non-motor symptoms, including tremors, cognitive deficits, sleep disturbance, and circadian rhythm experienced by patients with PD. A further closer look at the expression of other non-motor dysfunctions is warranted, as we have reported these motor and non-motor dysfunctions are faithfully modeled in the MPTP marmoset model. It is as important to consider the limitations of animal models in general. Nevertheless, it is essential to start rigorously considering these motor and non-motor dysfunctions as primary endpoints in translational endeavors for the development of better treatments for PD.

## Supporting information

S1 Video The video recording show typical low frequency resting tremor of the head of a parkinsonian marmoset. (MP4)

S2 Video The video recording show an animal falling asleep during daytime while engaged with the experimenter. (M4V)

S3 Video The video recording show baseline behavior of an animal reaching and retrieving a reward. After MPTP treatment, the same animal show Parkinson-like altered range of motion of the arm during reaching and significant improvement after L-DOPA treatment.
(MP4)
